# Anthelmintic resistance in gastrointestinal nematodes of small ruminants in Southern Africa: a review and comparative analysis

**DOI:** 10.1007/s11259-026-11351-9

**Published:** 2026-06-24

**Authors:** Songezo Mavundela, William Diymba Dzemo

**Affiliations:** Department of Biological and Environmental Sciences, Walter Sisulu University, Private Bag X1, Mthatha, 5117 South Africa

**Keywords:** Goat, Sheep, Gastrointestinal nematodes, Macrocyclic lactones, Benzimidazole, Anthelmintic resistance

## Abstract

Gastrointestinal nematodes (GINs) significantly limit small ruminant production in Southern Africa, causing substantial economic losses and reduced livestock productivity. Control largely depends on anthelmintic drugs, but intensive and improper use has accelerated the development of anthelmintic resistance (AHR). This review synthesizes published evidence on AHR in GINs of sheep and goats across Southern Africa, drawing on studies from six countries, and summarizes resistant parasite species, drug classes, diagnostic methods, and associated risk factors. Comparative analyses showed a significant association between anthelmintic class and the proportion of farms exhibiting resistance (*P* < 0.05). Macrocyclic lactone (ML) resistance, particularly to ivermectin, was most prevalent, with significantly more farms harbouring resistant GIN populations than susceptible ones (*P* < 0.05). In contrast, the proportions of farms showing resistance to benzimidazoles (BZ) and imidazothiazoles (IMD) did not differ significantly from those with susceptible populations, reflecting variability in resistance patterns at farm level. Salicylanilide compounds exhibited significantly lower resistance prevalence (*P* < 0.05). *Haemonchus* spp. was the dominant resistant genus, with 62.2% of farms reporting resistant populations, significantly exceeding those retaining susceptibility (*P* < 0.05). In contrast, lower resistance levels were observed in *Teladorsagia*, *Trichostrongylus*, *Oesophagostomum*, and *Cooperia* species. These findings indicate growing evidence of resistance to BZ, ML, and IMD compounds in GINs of small ruminants in Southern Africa. The underlying reasons are unclear but are likely driven by frequent whole-flock treatments, improper dosing, limited drug rotation, reduced refugia, cross-border livestock trade, communal grazing, and non-prescription use of anthelmintics.

## Background

Small ruminant farming plays a crucial role in the livelihoods of farmers in Southern Africa by providing protein and income through the sale of animals and wool (Goni et al. 2018; Kabinda et al. 2022). Livestock are kept as a form of investment, and for social purposes, including status, paying bride price, performing ancestral ceremonies, and providing manure in the region (Goni et al. 2018). However, despite their economic importance and social value, livestock farming is highly constrained by various limitations including lack of feed, poor infrastructure, lack of support from veterinary services, limited areas for grazing, lack of organized market access, water scarcity, treatment expenses, stock theft, diseases, and parasites that greatly reduce productivity and profitability (Eeswaran et al. 2022; Nontu et al. 2025). Parasitism due to gastrointestinal nematodes (GINs) have significantly hindered small ruminant production (Idris et al. 2019). Heavy GIN infections in sheep and goats result in reduced feed intake, diarrhoea, digestive disturbances, weight loss, compromised immunity, lower meat and wool quality, decreased fertility, anaemia, and high morbidity and mortality rates (Mthi and Nyangiwe 2018; Idris et al. 2019; Mahlehla et al. 2021). Furthermore, nematodes are responsible for disease complexes, production losses, and substantial economic repercussions to the livestock production (Tsotetsi et al. 2013). For instance, in South Africa (SA), GIN infections have been associated with annual sheep losses estimated at approximately USD 29 million, leading to significant declines in production (Mavundela et al. 2025a).

Farmers often rely on the use of anthelmintic drugs for the treatment and control of GIN infections in small ruminants (Tsotetsi et al. 2013; Haftu et al. 2020). Although anthelmintic drugs are known to improve animal health, their excessive and indiscriminate use has led to residues in animal products and the emergence of resistance (Tsotetsi et al. 2013; Wasso et al. 2020). Anthelmintic resistance (AHR) is a heritable trait in parasites that allows them to be less susceptible to the discriminating dose of a treatment. Over time, this trait becomes increasingly prevalent within a parasite population, leading to the development of resistance (Shalaby 2013; Belecke et al. 2021; Dzemo et al. 2022; Mavundela et al. 2025a). This phenomenon has been reported across the three major classes of anthelmintic drugs, including macrocyclic lactones (ML), imidazothiazoles (IMD), and benzimidazoles (BZ) (Nabukenya et al. 2014; Mavundela et al. 2025a). Multidrug resistance (MDR) is increasingly reported, in which a parasite population is able to withstand two or more anthelmintics with different modes of action (Fissiha and Kinde 2021). The estimated annual treatment costs for GIN infections are approximately USD 26 million in Kenya (Githiori et al. 2004), USD 81.8 million in Ethiopia (Asmare et al. 2016), USD 77.9 million in Nigeria (Odeniran et al. 2021), and USD 46 million in SA (Chagas et al. 2022).

The faecal egg count reduction test (FECRT) is commonly used to evaluate anthelmintic efficacy in small ruminants, in which egg counts before and after treatment are compared (Coles et al. 2006; Vatta and Lindberg 2006; Fissiha and Kinde 2021; Kaplan et al. 2023). When the FECRT results reveal reduced drug efficacy of < 95%, it indicates the development of AHR under field conditions (Emsley et al. 2023; Mphahlele et al. 2021). However, FECRT also measures treatment failure rather than resistance alone and may be influenced by factors such as dosing accuracy, drug quality, host immunity, and reinfection dynamics (Morgan et al. 2022). In addition, the FECRT has limited sensitivity for detecting emerging resistance when resistant parasites occur at low frequencies (< 25%), potentially leading to underestimation of early resistance (McIntyre et al. 2018; Morgan et al. 2022). Updated guidelines from the World Association for the Advancement of Veterinary Parasitology emphasise stricter study design requirements, including adequate sample sizes, confidence interval reporting, and standardised post-treatment sampling intervals, to improve the interpretation of FECRT results. However, these refinements do not fully overcome the essential limitations of the test for early resistance detection. Laboratory-based in vitro assays, including the egg hatch test (EHT), larval development tests, larval mortality assay (LMA), micro-agar larval development test (MALDT), and controlled efficacy test (an in vivo assay), are sometimes used to complement FECRT in the detection of resistance to anthelmintics under controlled conditions (Vatta and Lindberg 2006; Fissiha and Kinde 2021).

In Southern Africa, numerous reports of AHR in GINs of sheep and goats have been reported, in SA (Tsotetsi et al. 2013; Mphahlele et al. 2021; Mavundela et al. 2025a), Mozambique (Atanásio-Nhacumbe et al. 2017; Guinda et al. 2025), Tanzania (Bj∅rn et al. 1990; Keyyu et al. 2002; Changa and Kassuku 2006; Sailen et al. 2017), Zambia (Gabriel et al. 2001), Zimbabwe (Boersema and Pandey 1997), and Botswana (Ramabu et al. 2020). Some aspects of the AHR situation in South African small ruminant livestock, including helminth parasite prevalence and impact, as well as farmer awareness and knowledge, have been reviewed by Vatta and Lindberg (2006). Despite the availability of numerous published reports on AHR development in GINs of small ruminants in Southern Africa, no study has integrated findings from these studies to provide a comprehensive understanding of the nature and extent of the problem in the region. This is critical, as livestock trade, and interaction between treated and untreated livestock species are common within the region, promoting the spread of resistant GIN species between countries (Atanásio-Nhacumbe et al. 2017). This paper presents an overview and comparative analysis of the AHR situation in small ruminants of Southern Africa. The information provided is expected to inform future policies on managing the AHR problem and promoting strategies that reduce resistance and sustain anthelmintic efficacy in small ruminant production within the region.

### Search strategy and data analysis

This scoping review synthesized published evidence on AHR in GINs of sheep and goats. A structured literature search was conducted in PubMed, Web of Science, and Google Scholar, with no date restrictions. The final search was performed on 10 December 2025. Search terms were developed across four domains: host species, resistance concept, drug class/active ingredients, and parasite taxa. Keywords included: “sheep”, “goats”, “small ruminants”; “anthelmintic resistance”, “multidrug resistance”; “benzimidazole”, “albendazole”, “fenbendazole”, “oxfenbendazole”, “macrocyclic lactone”, “moxidectin”, “milbemycin”; and “gastrointestinal nematodes”, “roundworms”, “Nematodirus spp”., “Cooperia spp.”, “Haemonchus spp.”, “Oesophogostomum spp.”, and “Trichostrongylus spp.” Boolean operators (AND/OR) were used to construct database-specific search strings, which were adapted according to indexing structures. Due to platform limitations, combinations of the above terms were searched iteratively. Research list of included articles was manually examined to identify additional studies. Full texts were accessed through the Walter Sisulu University library databases. Only peer-reviewed articles published in English were included.

Titles and abstracts were screened for relevance to the study objectives. Studies were included if they reported primary field data on AHR in GIN of sheep and/or goats and provided extractable resistance outcomes. Reviews and studies lacking clear resistance metrics were excluded. Data extracted included: resistant GIN genera/species, anthelmintic class and active ingredient, diagnostic technique, reported risk factors, country of study, and proportion of farms exhibiting resistance. A standardized extraction template was used to ensure consistency.

As a scoping review incorporating quantitative elements, the primary objective was descriptive mapping of resistance patterns by country, animal host (sheep/goats), drug class/active ingredient, helminth species/genus, and detection method. Associations between: (i) anthelmintic class and resistance occurrence, and (ii) GIN genera/species and resistance occurrence were evaluated using a chi-square test of independence. Statistical significance was set at two-tailed *P* < 0.05. Where overall associations were significant, post-hoc pairwise comparisons of proportions were performed using a Bonferroni-adjusted z-test. Categories represented by fewer than five studies were excluded from inferential testing due to unreliable estimates and violation of chi-square assumptions (Higgins et al. 2024).

### Current anthelmintic resistance situation in Africa

There has been an increase in resistance development in GIN species populations in small ruminant farms to BZ, ML, and IDM, in many countries of Africa (Mphahlele et al. 2021; Mavundela et al. 2025a). The AHR problem has been reported from at least 15 countries, with most studies emanating from Ethiopia, Kenya, Uganda, and SA (Mohammedsalih et al. 2020; Mphahlele et al. 2021; Abbas and Hildreth 2022; Kalule et al. 2023; Mavundela et al. 2025a). The BZ compounds, including albendazole (ALBZ), fenbendazole (FBZ), thiabendazole (TBZ), and oxfendazole (OFZ), remain the most widely used anthelmintic drugs for GIN control (Byaruhanga 2013; Mphahlele et al. 2021; Wondimu and Bayu 2022). Macrocyclic lactones, including ivermectin (IVM), abamectin (ABN), and doramectin (DRN), and the IMD compounds, particularly levamisole (LEV) and tetramisole (TRL), have also been commonly used (Byaruhanga 2013; Wondimu and Bayu 2022).

Several GIN species have developed resistance to these anthelmintic drugs, including *Haemonchus* spp., *Trichostrongylus* spp., *Teladorsagia/Ostertagia* spp., *Strongyloides* spp., *Bunostomum* spp., *Nematodirus* spp., *Trichuris* spp., *Cooperia* spp., and *Oesophagostomum* spp. (Mohammedsalih et al. 2020; Mphahlele et al. 2021; Abbas and Hildreth 2022). A systematic review study of AHR in livestock in Africa has noted that the prevalence of AHR is highly heterogeneous with considerable variation in GIN species, anthelmintic classes, and livestock species (Gatitu et al. 2025). In Ethiopia, 100% of sheep farms showed reduced ALBZ efficacy, with strongyle species demonstrating resistance (Molla et al. 2023). Mohammedsalih et al. (2019) noted BZ resistance in *H. contortus* on 66% of goat farms in South Darfur, Sudan. *Haemonchus contortus* resistance to ALBZ has been detected in sheep farms in Egypt (Aboelhadid et al. 2021). Byaruhanga and Okwee-Acai (2013) reported resistance to ALBZ in *Haemonchus* spp., *Cooperia* spp., and *Oesophagostomum* spp. in a goat farm in Uganda. Reduced efficacy of IVM against *H. contortus*, *Trichostrongylus colubriformis*, and *Oesophagostomum* spp. was observed on a goat farm in Nigeria (Idika et al. 2022). Resistance to IVM in *Trichostrongylus* spp., *Haemonchus* spp., and *Oesophagostomum* spp. was detected in two sheep farms of Nziih, West Cameroon (Stephanie et al. 2022).

Multidrug AHR in GINs of small ruminants has also been reported in several countries, including Ethiopia (Kumsa and Abebe 2009; Wondimu and Bayu 2022), Kenya (Waruiru et al. 1998), Uganda (Byaruhanga and Okwee-Acai 2013), and Algeria (Bentounsi et al. 2007). The presence of *Haemonchus* spp. resistant to ALBZ and FBZ was noted on a sheep farm in eastern Algeria (Bentounsi et al. 2006). Bentounsi et al. (2007) also detected ALBZ and IVM resistance in *Teladorsagia* spp. and *Trichostrongylus* spp. on 35% and 7% of sheep farms, respectively, in the eastern part of Algeria. In the Gomba District of Uganda, resistance to ALBZ, LEV, and IVM occurred in *Haemonchus* spp. on 100% of goat farms (Nsereko et al. 2013). Furthermore, *Haemonchus* spp. resistant to IVM, LEV, and ALBZ was detected in 58%, 52%, and 38% of sampled goat farms, respectively, from Soroti, Gulu, Mpigi, Mbarara, and Ssembabule districts (Nabukenya et al. 2014). In Kenya, Maingi (1991) identified BZ and LVM resistance in *Haemonchus* spp. and *Trichostrongylus* spp. on a private sheep farm in Karen. In Diani Estate, Kenya, Mwamachi et al. (1995) reported that *H. contortus*, *Trichostrongylus* spp., and *Oesophagostomum* spp. were resistant to IVM and FBZ on both sheep and goat farms. Another study in Kenya reported resistance of *Haemonchus* spp. and *Trichostrongylus* spp. to both BZ and LEV in sheep and goat farms in the Eldoret, Karatina, Kericho, Nakuru, and Mariakani regions (Wanyangu et al. 1996). Resistance in *H. contortus* to BZ, LEV, and IVM was also detected in a sheep farm in north-western Nairobi (Waruiru et al. 1998). In Ethiopia, *Haemonchus contortus* resistant to ALBZ, IVM, and tetramisole was reported in Dorper sheep farms in the Digeluna Ticho and Wuchale Districts (Eguale et al. 2010). Additionally, Wondimu and Bayu (2022) noted *Trichostrongylus* spp. and *Teladorsagia* spp. resistant to both IVM and ALBZ, as well as *Haemonchus* spp. resistant to tetraclozan in the Haramaya District. The use of stubble fields, which reduces refugia, along with frequent treatments, particularly with BZ compounds, has been identified as a major driver of resistance in small ruminant farming systems (Wanyangu et al. 1996; Maingi 1991; Bentounsi et al. 2007). Underdosing, inaccurate weight estimation, and unregulated drug purchase, have been strongly associated with decreased drug efficacy (Nsereko et al. 2013; Byaruhanga and Okwee-Acai 2013). The communal grazing systems, high stocking density, and uniform treatment strategies across neighbouring smallholder farms have also been associated with the issue of anthelmintic control failure (Wondimu and Bayu 2022; Molla et al. 2023). A closer attention to these risk factors could reduce selection pressure and improve productivity in small ruminant production systems.

### Spatiotemporal patterns of anthelminthic resistance in Southern Africa

A total of 26 studies, comprising 73 bioassays, assessed AHR in the Southern African region (Table 1). Reports of resistance to BZ, ML, IMD, and salicylanilides (SLD), have been documented in six of thirteen Southern African countries (Boerserma and Pandey 1997; Keyyu et al. 2002; Bakunzi 2003; Ramabu et al. 2020; Mavundela et al. 2025a) (Table 1), with the majority of studies from SA and Tanzania (Table 2). In Tanzania, all seven studies encompassing 13 bioassays reported AHR in *Haemonchus* spp. and *Trichostrongylus* spp. to BZ, ML, and IDM, on 37 sampled farms (Bjørn et al. 1990; Msangi et al. 1990; Ngomuo et al. 1990; Keyyu et al. 2002; Changa and Kassuku 2006; Mirambo and Kassuku 2008; Sailen et al. 2017) (Table 2). No published data were identified from Angola, Comoros, Eswatini, Lesotho, Madagascar, Malawi, Mauritius, and Namibia. This surveillance gap likely reflects limited awareness, veterinary infrastructure, and technical capacity (World Organisation for Animal Health 2021), and the high costs associated with research and implementation of effective surveillance tools (Nixon et al. 2020).

**Table 1:** Studies reporting anthelmintic resistance in small ruminants in Southern Africa

Country	Animal	Anthelmintic drug (farms with resistance/ farms sampled)	Anthelmintic drug (% reduction)	Helminth species	Detection method	Reference
South Africa	Sheep	-	Parbendazole (79.40)	*H. contortus*	Controlled efficacy test	Berger (1975)
			Thiabendazole (37.50)	*H. contortus*		
			Cambendazole (66.40)	*H. contortus*		
			Mebendazole (63.00)	*H. contortus*		
			Fenbendazole (80.80)	*H. contortus*		
	Sheep	-	Rafoxanide (65.50)	*H. contortus* (Ptzr strain)	Controlled efficacy test	Van Wyk et al. (1987)
			Fenbendazole (44.50)	*H. contortus* (Ptzr strain)		
			Rafoxanide (13.90)	*H. contortus* (Krtz strain)		
			Fenbendazole (0.00)	*H. contortus* (Krtz strain)		
	Sheep	Levamisole (6/9)	-	*H. contortus*	FECRT	Van Wyk et al. (1999)
		Rafoxanide (3/12)	-	*H. contortus*		
		Albendazole (6/12)	-	*H. contortus*		
		Ivermectin (2/6)	-	*H. contortus*		
	Goats	Albendazole (7/12)	-	*H. contortus*		
		Ivermectin (5/6)	-	*H. contortus*		
	Sheep	Albendazole (9/15)	-	GIN species not specified	FECRT	Dreyer (2002)
		Levamisole (6/15)	-	GIN species not specified		
		Ivermectin (3/15)	-	GIN species not specified		
	Goat	Rafoxanide (3/10)	-	GIN species not specified	FECRT	Bakunzi (2003)
		Levamisole (2/10)	-	GIN species not specified		
		Fenbendazole (1/10)	-	GIN species not specified		
	Sheep	Albendazole (1/12)	-	GIN species not specified	FECRT	Bakunzi (2008)
		Levamisole (4/12)	-	GIN species not specified		
		Closantel (1/12)	-	GIN species not specified		
	Sheep	Albendazole (1/10)	-	GIN species not specified	FECRT	Bakunzi et al. (2013)
		Ivermectin (1/10)	-	GIN species not specified		
		Closantel (4/10)	-	GIN species not specified		
	Goat	Albendazole (4/10)	-	GIN species not specified		
		Ivermectin (6/10)	-	GIN species not specified		
		Closantel (4/10)	-	GIN species not specified		
	Sheep	Ivermectin (3/3)	-	*Haemonchus* spp., *Teladorsagia/Trichostrongylus* spp.	FECRT	Tsotetsi et al. (2013)
		Albendazole (2/3)	-	*Haemonchus* spp., *Teladorsagia/Trichostrongylus* spp.		
		Levamisole (2/3)	-	*Haemonchus* spp., *Teladorsagia/Trichostrongylus* spp.		
	Goats	Ivermectin (2/2)	-	*Haemonchus* spp., *Teladorsagia/Trichostrongylus* spp.		
		Albendazole (2/2)	-	*Haemonchus* spp.		
		Levamisole (2/2)	-	*Haemonchus* spp.		
	Sheep	Albendazole (4/5)	-	*H. contortus*	FECRT	Mphahlele et al. (2021)
		Levamisole (3/5)	-	*H. contortus*		
		Macrocyclic lactones (3/5)	-	*H. contortus*		
	Sheep	Trichlorfon (1/1)	-	*H. contortus*	FECRT	Van der Merwe (2021)
		Closantel + albendazole (1/1)	-	*H. contortus*		
		Ivermectin (1/1)	-	*H. contortus*		
		Levamisole (1/1)	-	*H. contortus*		
	Sheep	Albendazole (3/3)	-	*Haemonchus* spp.	FECRT	Emsley et al. (2023)
		Levamisole (3/3)	-	*Haemonchus* spp.		
		Ivermectin (3/3)	-	*Haemonchus* spp.		
	Sheep	Albendazole + Closantel (6/6)	-	*Haemonchus* spp.	FECRT	Mavundela et al. (2025a)
		Levamisole hydrochloride (3/3)	-	*Haemonchus* spp.		
		Ivermectin (6/6)	-	*Haemonchus* spp.		
		Levamisole + Praziquantel (1/1)	-	*Haemonchus* spp.		
Mozambique	Goats	Albendazole (2/12)	-	*Haemonchus* spp. *Trichostrongylus* spp. *Oesophagostomum* spp.	FECRT	Atanasio and Sitoe (2002)
		Fenbendazole (2/12)	-	*Haemonchus* spp. *Trichostrongylus* spp. *Oesophagostomum* spp.		
		Levamisole (4/12)	-	*Haemonchus* spp. *Trichostrongylus* spp. *Oesophagostomum* spp.		
	Goats	Albendazole (8/11)	-	*Haemonchus* spp.	FECRT	Atanásio-Nhacumbe et al. (2017)
	Goats	Albendazole (8/12)	-	*H. contortus*	FECRT	Atanásio-Nhacumbe et al. (2019)
	Goats	Fenbendazole (5/16)	-	*H. contortus* *T. colubriformis*	FECRT	Guinda et al. (2025)
Tanzania	Sheep	Albendazole (1/1)	-	*H. contortus*	FECRT	Bjørn et al. (1990)
	Sheep	-	Fenbendazole (0%)	*H. contortus* (DASP strain)	Controlled efficacy test	
			Fenbendazole (70.50%)	*H. contortus* (MKATA strain)		
	Goat	-	Oxfendazole (15%)	*H. contortus* (SUA strain)	Controlled efficacy test	Msangi et al. (1990)
	Goats	-	Thiophanate (24%)	*H. contortus* (SUA strain)	Controlled efficacy test	Ngomuo et al. (1990)
	Sheep	Albendazole (1/1)	-	*H. contortus*	FECRT	Keyyu et al. (2002)
	Sheep	Albendazole (8/8)	-	*Haemonchus* spp. *Trichostrongylus* spp.	FECRT	Changa and Kassuku (2006)
		Levamisole+ oxyclozanide (8/8)	-	*Haemonchus* spp. *Trichostrongylus* spp.		
		Ivermectin (8/8)	-	*Haemonchus* spp. *Trichostrongylus* spp.		
	Goat	Fenbendazole (1/1)	-	*Haemonchus* spp.	FECRT	Mirambo and Kassuku (2008)
		Levamisole (2/2)	-	*Haemonchus* spp.		
	Sheep	Fenbendazole (1/1)	-	*Haemonchus* spp.		
		Levamisole (2/2)	-	*Haemonchus* spp.		
	Goat	Albendazole (1/1)	-	*H. contortus*	FECRT	Sailen et al. (2017)
		Levamisole (1/1)	-	*H. contortus*		
	Sheep	Albendazole (1/1)	-	*H. contortus*		
		Levamisole (1/1)	-	*H. contortus*		
Zimbabwe	Sheep	Albendazole (4/5)	-	*Haemonchus* spp. *Cooperia* spp.	FECRT	Mukaratirwa et al. (1997)
		Oxfenbendazole (2/3)	-	*Haemonchus* spp.		
		Fenbendazole (1/1)	-	*Haemonchus* spp.		
		Levamisole (1/3)	-	*Haemonchus* spp.		
	Sheep	Fenbendazole (7/10)	-	*H. contortus*	FECRT	Boersema and Pandey (1997)
		Levamisole (4/10)	-	*H. contortus*		
		Rafoxanide (6/10)	-	*H. contortus*		
	Sheep	Ivermectin (1/1)	-	*Haemonchus* spp. *Cooperia* spp.	FECRT	Mushonga et al. (2024)
		Albendazole (1/1)	-	*Haemonchus* spp.		
		Levamisole (1/1)	-	*Haemonchus* spp. *Cooperia* spp.		
Zambia	Sheep	Albendazole (5/6)	-	*Haemonchus* spp.	FECRT	Gabriel et al. (2001)
		Ivermectin (2/6)	-	*Haemonchus* spp.		
Botswana	Goats	Ivermectin (2/10)	-	GIN species not specified	FECRT	Ramabu et al. (2020)

Abbreviations: *BZ*, benzimidazoles; *BZ + SLD*, co-formulation of benzimidazoles and salicylanilides; *ISN*, isoquinoline; *ISN + IMD*, co-formulation of imidazothiazoles+isoquinoline; *SLD*, salicylanilides; *ML*, macrocyclic lactones

**Table 2:** Geographic distribution and prevalence of anthelmintic resistance on small ruminant farms in Southern Africa

Country	No of studies	No of bioassays	Proportions of resistance	
			Proportion of farms exhibiting resistance	Prevalence of farms with resistance (%)
South Africa	10	41	131/287	45.64
Mozambique	4	6	29/75	38.67
Tanzania	7	13	37/37	100.00
Zimbabwe	3	10	28/45	62.22
Zambia	1	2	7/12	58.33
Botswana	1	1	2/10	20.00

**Fig. 1:**
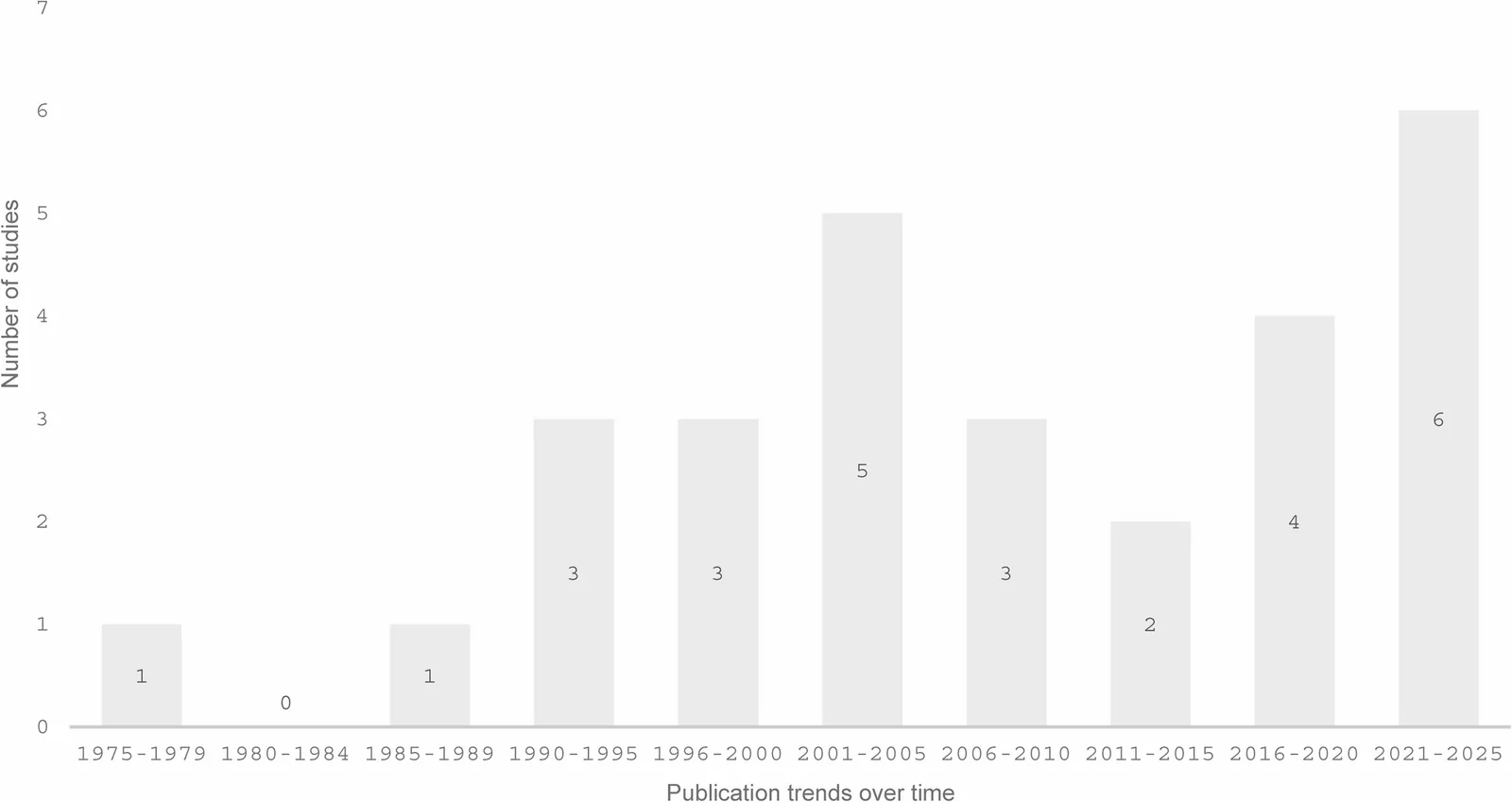
Yearly trends in resistance development in gastrointestinal nematode populations of small ruminants in Southern Africa

The first documented case of nematode resistance in sheep was reported in South Africa in 1975, where a controlled efficacy test revealed that *Haemonchus contortus* had developed cross-resistance to five benzimidazole compounds (TBZ, parbendazole, cambendazole, mebendazole, and FBZ) (Berger 1975). Since then, a gradual increase in resistance among GIN populations of small ruminants has been observed (Fig. 1). This temporal rise reflects the cumulative selection pressure from prolonged anthelmintic use, which promotes the survival and reproduction of resistant worms and facilitates the progressive fixation of resistance alleles within GIN populations (Abbott et al. 2012; Rychlá et al. 2024).

Evidence on AHR development in GINs of small ruminant farms in the Southern African region indicates a significant association between anthelmintic drug class and the proportion of farms exhibiting resistance, (* x*^2^_(3, *N* = 493)_ = 25.54, *P* < 0.001). The proportions of farms with ML (IVM) resistant-GIN species populations are significantly higher (*P* < 0.05) than those where populations remained susceptible (Table 3). Several studies in Southern Africa have documented resistance of GINs, particularly *H. contortus*, to ML such as IVM. In South Africa, reduced efficacy of IVM has been reported on sheep farms in the Western Cape (Dreyer 2002), Gauteng (Tsotetsi et al. 2013; Limpopo (Mphahlele et al. 2021), and the Eastern Cape (Mavundela et al. 2025a), often occurring alongside resistance to other anthelmintic classes. Similar patterns have been reported elsewhere in the region, including Zimbabwe (Mushonga et al. 2024), Zambia (Gabriel et al. 2001), and Botswana (Ramabu et al. 2020), highlighting the increasing prevalence of ML resistance in small ruminant production systems and its implications for sustainable parasite control. Resistance to ML is firmly established in small ruminant production systems worldwide, with resistance levels exceeding 50% on sheep and goat farms across Australia, Europe, and South America (Kaplan and Vidyashankar 2012). In Australia, IVM resistance was detected on 67% of surveyed sheep farms in a temperate region of Victoria, with higher resistance proportions than those reported for other ML compounds (abamectin and moxidectin) (Preston et al. 2019). In South America, Batista et al. (2022) documented widespread IVM resistance on sheep farms in the semiarid region of Minas Gerais, Brazil, with no effective faecal egg count reduction observed across 17 surveyed flocks. In Europe, IVM resistance was detected in 78.3% of surveyed sheep flocks in the Netherlands, indicating widespread loss of ML efficacy against GINs (Ploeger and Everts 2018). Macrocyclic lactones, particularly IVM, were widely adopted globally in 1981, soon after their introduction, due to their reliability under extensive and semi-extensive grazing systems, ease of administration, and perceived superiority over older compounds (FAO 2004). Macrocyclic lactones have a wide spectrum of activity, being used not only against helminths (nematodes) but also against arthropods (acarids and insects) (Tsotetsi et al. 2013; FAO 2025). Their use in controlling ectoparasites on small ruminants has further exacerbated the development of resistance in GINs, as systemic absorption of IVM, particularly from pour-on formulations, results to prolonged exposure of nematodes to sub-therapeutic drug concentrations (Geurden et al. 2015; Vercruysse et al. 2018). Such exposure preferentially eliminates susceptible genotypes while allowing partially resistant individuals to survive and reproduce, thereby increasing the frequency of resistance alleles in the population (Kaplan and Vidyashankar 2012).

**Table 3:** Prevalence of anthelmintic resistance on small ruminant farms in Southern Africa

GIN species	Anthelmintic drug class and proportion of farms with resistance											
	BZ	IMD	ML	SLD	BZ + SLD	IMD + ISN	IMD + SLD	No. of studies	Total proportions	Prevalence of farms with resistant GIN genera/ species (%)	Prevalence of farms with susceptible GIN genera/ species (%)	𝑥^2^- test p-value
Haemonchus spp.	73/126	30/52	33/40	9/22	6/6	1/1	8/8	18	156/251	62.20a	37.80b	< 0.001
*Not specified	16/17	12/37	10/35	12/42	-	-	-	5	50/131	38.17	61.83	
*Trichostrongylus spp.	20/51	6/15	13/13	-	-	-	8/8	3	47/87	-	-	
*Teladorsagia spp.	3/3	2/3	5/5	-	-	-	-	1	10/11	-	-	
*Oesophagostomum spp.	4/24	4/12	-	-	-	-	-	1	8/36	-	-	
Cooperia spp.	4/5	-	-	-	-	-	-	3	4/5			
No of studies included	18	13	10	5	1	1	1	-	-	-	-	
Total proportion with resistance	118/224	53/118	60/92	21/64	6/6	1/1	16/16	-	-	-	-	
Prevalence of farms with resistance (%)	52.40a	46.70a	73.20a	32.80a	-	-	-	-	-	-	-	
Prevalence of farms with susceptibility (%)	4 7.60a	53.30a	26.80b	67.20b	-	-	-	-	-	-	-	
𝑥^2^- test p-value	<0.001				-	-	-	-	-	-	-	

Similar lowercase letters denote resistance status for anthelmintic drug classes/ GIN genera whose total proportions (%) do not differ significantly (*P* > 0.05).

^*^Assessment of associations between proportions was withheld in categories represented by fewer than five studies, as statistical comparisons become unreliable when study numbers are low. (Higgins et al. 2024).

Abbreviations: *BZ*, benzimidazoles; BZ+SLD, co-formulation of benzimidazoles and salicylanilides; *ISN*, isoquinoline; ISN+IMD, co-formulation of imidazothiazoles+isoquinoline; *SLD*, salicylanilides; *ML*, macrocyclic lactones.

This study further noted that the proportion of farms exhibiting IMD and BZ resistance in the Southern African region did not differ significantly (*P* > 0.05) from those harbouring susceptible GIN populations within the Southern African region (Table 3). Surveys in SA reported BZ and IMD resistance on some farms but not others within the same production areas, reflecting variability in resistance patterns at the farm level (Bakunzi 2003; Tsotetsi et al. 2013; Mphahlele et al. 2021). Similarly, studies in Zimbabwe and Zambia detected resistance to BZ and IMD on several farms while other farms remained susceptible (Boersema and Pandey 1997; Gabriel et al. 2001). This heterogeneous distribution of resistance across farms supports the observation reported in this study. It aligns with findings from previous global surveys, which have demonstrated that resistance to BZ and LEV tends to be unevenly distributed across farms rather than occurring as a uniformly high prevalence. Traversa and von Samson-Himmelstjerna (2016) documented BZ- and LEV-resistant gastrointestinal strongyles in several European countries, highlighting considerable variability in resistance patterns and the continued coexistence of resistant and susceptible parasite populations. In Australia, Overend et al. (1994) conducted a large-scale survey of 881 sheep farms and noted a widespread resistance to BZ and LEV in GINs, with only a minority of farms achieving ≥ 95% efficacy after treatment. A field study in the São Luís metropolitan area of Maranhão, Brazil, found reduced anthelmintic efficacy against gastrointestinal strongyles in small ruminants, with LEV producing 0% faecal egg count reduction in goats and 79.4% in sheep, while BZ (ALBZ) achieved 59.8% efficacy in goats and only 3.43% in sheep (Soares et al. 2023). Globally, control strategies for GINs remain heavily reliant on the use of broad-spectrum anthelmintics, particularly BZ, IMD, and ML (Potârniche et al. 2021; Gatitu et al. 2025; Mavundela et al. 2025a). The absence of a significant difference between BZ-and IMD-resistant and susceptible farm proportions may reflect variability in anthelmintic usage patterns, refugia management, and dosing accuracy, all of which strongly influence resistance development at the farm level (Van Wyk and Rayneck 2011). Moreover, phenotypic assessment of AHR using the FECRT is poor at detecting early or low levels of resistance, which may have contributed to the apparent parity between resistant and susceptible GIN populations (Coles et al. 2006).

The proportion of small ruminant farms in the Southern African region on which GIN populations exhibited resistance to SLD chemical compounds was significantly lower (*P* < 0.05) than the proportion of farms on which GIN populations remained susceptible (Table 3). This finding is consistent with a European report by Claerebout et al. (2020), which documented widespread treatment failure with BZ and ML on sheep farms in Belgium. In contrast, SLD (closantel) failure was detected on only a single farm, suggesting comparatively preserved efficacy of SLDs under field conditions. Additionally, on an organised goat farm in the Kashmir Valley, India, closantel (CLO) reportedly retained moderate efficacy relative to fenbendazole and IVM (Bihaqi et al. 2020). In the Southern American region, a controlled efficacy study conducted in Uruguay demonstrated that CLO, particularly when co-administered with moxidectin, achieved high efficacy against multidrug-resistant GINs in lambs (Suárez et al. 2023). This pattern may reflect a comparatively lower, more targeted use of SLDs compared to BZs, IMDs, or MLs, which are known to exhibit widespread resistance in small ruminant production systems in Southern Africa. Additionally, SLDs have a narrow spectrum and are often used less frequently or in specific management scenarios, such as targeted treatments of blood-feeding parasites (*Haemonchus* spp.) or as combination therapies, which may further reduce the sustained selection pressure for resistance, allowing susceptibility to remain relatively higher on many farms (Lanusse et al. 2015; Kaplan and Vidyashankar 2012). However, despite the observed low prevalence of resistance to SLD compounds, the presence of resistant GIN populations on some farms indicates that resistance development is possible and ongoing.

### Genus–level patterns of anthelmintic resistance in gastrointestinal nematodes

Within the Southern African region, AHR was most frequently detected in *Haemonchus* spp., whereas substantially lower levels were observed in *Teladorsagia* spp., *Trichostrongylus* spp., *Oesophagostomum* spp., and *Cooperia* spp. (Table 3). This genus-specific pattern of resistance is consistent with previous studies from Southern Africa and other small ruminant production systems where *H. contortus* has repeatedly been identified as the main contributor to AHR (Van Wyk et al. 1999; Kaplan 2004; Van Wyk and Reynecke 2011; Mavundela et al. 2025a). The significantly higher proportions of farms reporting anthelmintic-resistant *Haemonchus* spp. (62.20%) compared with farms where susceptibility was preserved (*P* < 0.05) highlights the central role of this genus in shaping regional resistant patterns. The propensity of *Haemonchus* spp. to develop resistance has been attributed to their high fecundity (females producing 5000–10000 eggs per day under favourable conditions), and short life cycle (about 18–21 days) (Abbott et al. 2012; Rychlá et al. 2024). Furthermore, its ability to exploit favourable climatic conditions, such as the warm and humid environments characteristic of much of Southern Africa, supports a year-round transmission and frequent anthelmintic intervention (Kaplan 2004). In contrast, the comparatively lower resistance levels observed in *Teladorsagia* spp., *Trichostrongylus* spp., *Oesophagostomum* spp., and *Cooperia* spp., may reflect differences in effective drug exposure due to their seasonal epidemiology, including lower egg production (100 to 3000 eggs per day), seasonal transmission, greater maintenance of refugia population, and lower dominance within mixed-species infections (Van Wyk et al. 2006; Shalaby 2013; Hou et al. 2022; Macedo et al. 2023; Otranto and Wall 2024). Furthermore, FECRT, which is the commonly used phenotypic diagnostic tool, is sensitive to changes in *Haemonchus* spp., egg output potentially increasing the likelihood of detecting resistance in this genus (Coles et al. 2006).

### Factors and drivers of anthelmintic resistance in Southern Africa

A key driver of AHR in the region is the frequent and prolonged use of a single anthelmintic class with minimal rotation. In several South African provinces, repeated reliance on BZ and ML compounds has selected for MDR *Haemonchus contortus* populations (Van der Merwe 2021; Mavundela et al. 2025a). Similar trends were reported in Mozambique, where albendazole resistance in *Haemonchus*,* Oesophagostomum*, and *Trichostrongylus* spp. expanded markedly between 2002 and 2017 (Atanásio et al. 2002; Atanásio-Nhacumbe et al. 2017). In Tanzania, prolonged BZ use on institutional farms led to persistent resistance, with no reversion to susceptibility even a decade after drug withdrawal (Bjørn et al. 1990; Keyyu et al. 2002; Mirambo and Kassuku 2008). These observations align with global evidence that once resistance becomes established, it is often irreversible due to the stability of resistance alleles within parasite populations (Kaplan and Vidyashankar 2012). A second major driver of AHR is inaccurate dosing, often resulting from failure to weigh animals and reliance on visual estimates. In the North West Province of SA, underdosing was linked to resistance to ALBZ, LEV, and CLO in communal systems (Bakunzi 2003, 2008; Bakunzi et al. 2013). Similar patterns occurred in Gauteng and Limpopo, where incorrect dosing, lack of weighing equipment, and repeated use of the same compounds promoted resistance in *Haemonchus*, *Teladorsagia*, and *Trichostrongylus* spp. (Tsotetsi et al. 2013; Mphahlele et al. 2021). Underdosing allows heterozygous resistant worms to survive, gradually increasing the frequency of the resistance allele over successive generations (Suarez 2002; Vatta and Lindberg 2006).

Animal movement and the introduction of resistant genotypes are additional key drivers of AHR. In communal and emerging farming systems, stock is frequently purchased from commercial farms or across borders without quarantine or pre-treatment. In Mozambique, imported Boer and Kalahari Red goats introduced albendazole-resistant *H. contortus* populations (Atanásio-Nhacumbe et al. 2017), while in Zambia, sheep imports from Zimbabwe likely facilitated regional dissemination of BZ-resistance strains (Gabriel et al. 2001). Limited post-introduction surveillance across the region has allowed the silent spread of resistant nematode strains (Ramabu et al. 2020). Management practices that reduce refugia have further exacerbated the AHR. In the Free State and Eastern Cape provinces of South Africa, MDR *H. contortus* populations were associated with misuse of broad-spectrum drugs and inadequate refugia maintenance (Van der Merwe 2021; Mavundela et al. 2025b). Intensive whole-flock treatments eliminate susceptible worms, leaving predominantly resistant parasites to repopulate pastures (Kaplan and Vidyashankar 2012). Given that resistance alleles may exhibit dominant inheritance, rapid selection occurs when refugia are insufficient (Babják et al. 2023).

Substandard or counterfeit drugs, along with inappropriate application techniques, have been reported in Tanzania and other parts of the region (Keyyu et al. 2002; Sailen et al. 2017). Poor-quality formulations can deliver sub-therapeutic concentrations, compounding the impact of inaccurate dosing. In communal systems, economic constraints often drive the use of cheaper compounds, such as levamisole or albendazole, intensifying class-specific selection pressure (Bakunzi 2008; Tsotetsi et al. 2013). Finally, limited surveillance and fragmented monitoring constrain early detection and mitigation of AHR. A recent continental review found that only 28 of 357 African studies met criteria for AHR evaluation, highlighting major knowledge gaps (Gatitu et al. 2025). Under-reporting likely masks the true extent of resistance, particularly in Zimbabwe, Zambia, and Botswana, where sporadic reports indicate established resistance, but systematic data remain scarce (Boersema and Pandey 1997; Mukaratirwa et al. 1997; Gabriel et al. 2001; Mushonga et al. 2024). Multifactorial drivers, including underdosing, frequent whole-flock treatments, minimal drug rotation, reduced refugia, unmonitored animal movement, substandard drugs, and limited surveillance, have promoted AHR in GINs across Southern Africa, threatening small ruminant productivity. Urgent implementation of evidence-based, integrated parasite management is essential to preserve drug efficacy and safeguard regional food security (Fissiha and Kinde 2021; Mavundela et al. 2025a).

A key limitation of this review is that a keyword-based search strategy may have omitted relevant studies using alternative terminology or taxonomic classification. Comparisons of GINs resistance proportions across anthelmintic classes are further constrained by substantial methodological heterogeneity among the included studies, particularly inconsistencies in the application and interpretation of the FECRT. The predominance of this phenotypic assay, together with limited molecular evidence, may have led to underestimation of resistance in some settings. Uneven regional representation and potential publication bias are also possible, as studies reporting confirmed resistance may be more likely to be published than those demonstrating susceptibility. Despite these constraints, the structured search strategy and transparent analytical framework employed provide a comprehensive mapping of AHR patterns in small ruminant GIN in Southern Africa. The findings highlight consistent and recurring resistance trends that underscore the need for continued surveillance and standardize monitoring approaches.

## Conclusion

This regional synthesis provides a comprehensive overview of AHR in small ruminant GINs across Southern Africa, moving beyond individual-country studies to reveal broader spatiotemporal, drug-class, and genus-specific patterns. This integrated multi-country dataset highlights the pervasive prevalence of ML resistance, the heterogeneous distribution of BZ and IMD (LEV) resistance across farms, and the comparatively preserved efficacy of SLD. The review also exposes critical surveillance gaps, particularly in Zimbabwe, Zambia, and Botswana, emphasizing the need for coordinated cross-border monitoring systems to manage the spread of resistant nematodes driven by livestock movement or trade. These findings provide a scientific basis for regionally harmonized strategies, including improved dosing protocols, targeted refugia management, and rational anthelmintic rotation, essential in preserving drug efficacy and small ruminant productivity across the region.

## Data Availability

The datasets generated during and/or analysed during the current study are available from the corresponding author upon reasonable request.
